# Supplementary figures and images for: Psoralidin induces autophagy through ROS generation which inhibits the proliferation of human lung cancer A549 cells
Source: PeerJ. 2014 Sep 9;2:e555. doi: 10.7717/peerj.555 (PMC4168759; doi:10.7717/peerj.555)

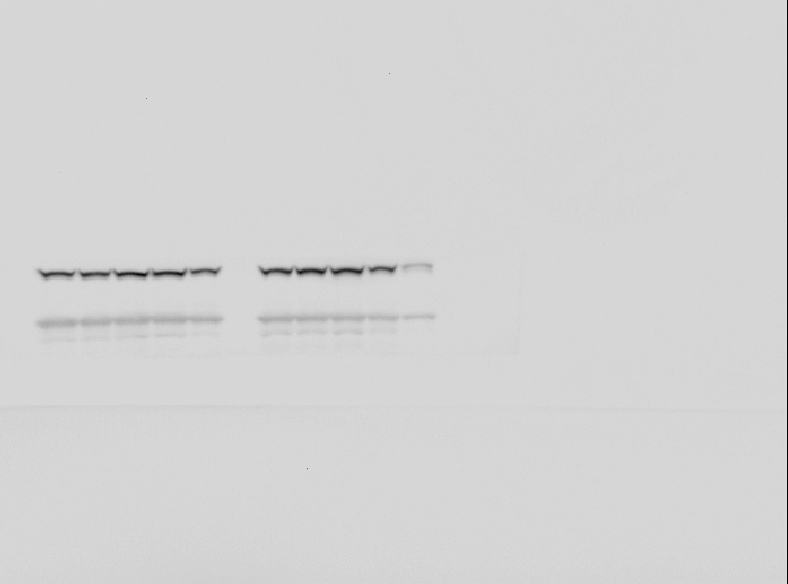

Supplement: Supplemental Information 1 [file peerj-02-555-s001.jpg]

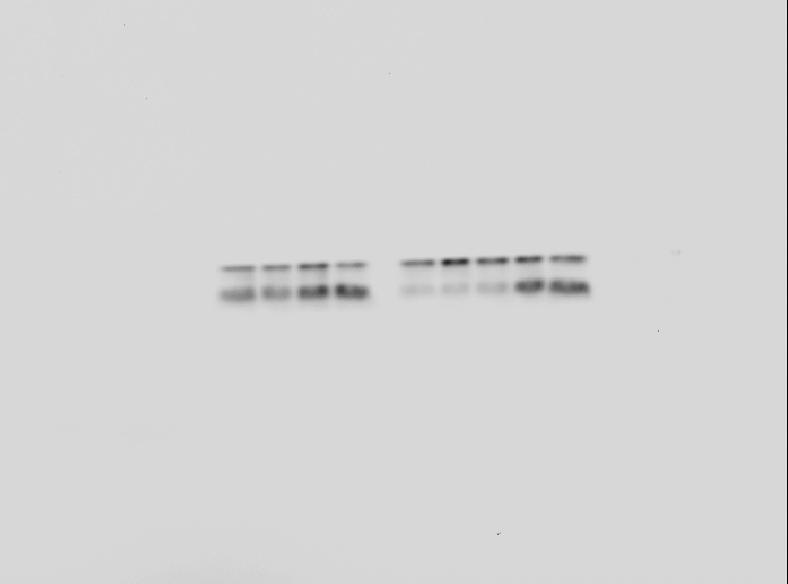

Supplement: Supplemental Information 2 [file peerj-02-555-s002.jpg]

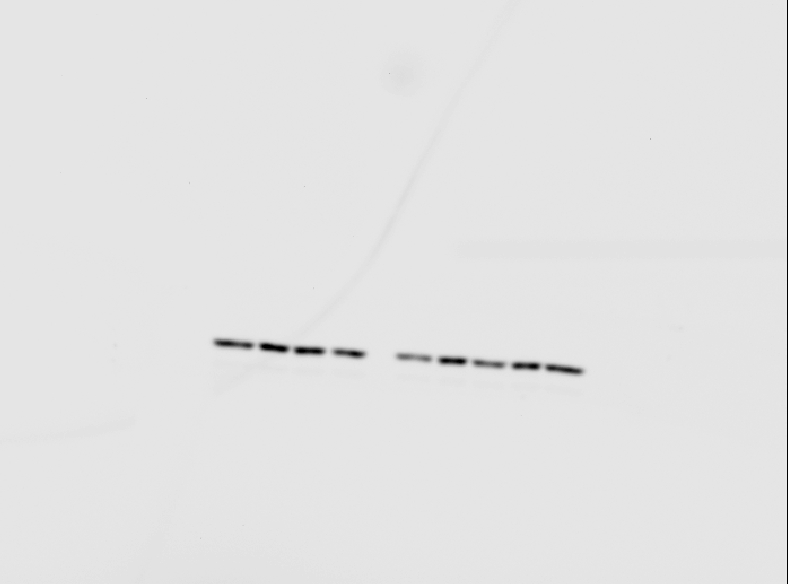

Supplement: Supplemental Information 3 [file peerj-02-555-s003.jpg]
